# The Child's Psychology

**Published:** 1920-10-16

**Authors:** 


### THE PUBLIC WELL-BEING.

### The Child's Psychology.

### DANGERS OF CRUDE MELODRAMA.

The relation of children and cinemas has been discussed from various angles recently (see page 44), and each one of them needs far closer consideration, both by the authorities and the cinematograph producers, than has yet been given. There are the questions of proper ventilation, of proper hours of attendance, of proper pictures for children to see, and also of employment of children in film studios. Each is important, for the cinema now forms so large a part of the entertainment of the people—and especially of children—and it has so definite an effect upon the social and psychological life of the country, that every effort should be made to see that its influences in all directions shall have all that is vitiating eliminated from them. The subject of proper accommodation and ventilation is one which can easily be controlled by the authorities acting upon expert advice. The matter of the hours of attendance by children is not so easy, but it is common knowledge that infants and young children are nearly always to be found in heavily smoke-laden cinema houses during the late night exhibitions, and no one can suppose that anything but harm arises.

#### The Kind of Picture.

What is equally vital is the kind of picture shown. No wise parent would take a child to a cinema without having first witnessed the programme. On the whole it would not be just to criticise the moral standard of the exhibitions, which is probably superior to the music-halls. It is not often that one sees anything that is objectionable in that sense. But much harm can be done, and must be done, to children by the predominance of crude melodramas, most of them of American origin. Here is a brief summary of a particular film shown last week in a good residential suburb of London. It opens with a fierce storm and shows a terrified woman looking through the window. Her facial agony, of which the producer seems vastly proud, is depicted again and again during the first half of the story. There is a wreck, and the audience is shown in detail the withdrawal of a dead man from the sea, his conveyance to the cottage, and his laying out on a bed, the woman's terrified features being prominently introduced—solo—at every opportunity. Then follows a funeral, an emotional confession of approaching motherhood by the unmarried woman, and the violence of her puritanical brother. In time this woman's suicide is shown on the screen; there is another scene where a man is killed in a brawl, one where a ruffian assaults a girl, a scene of temporary madness and horror, and another death, until finally, the rest live happily ever after.

#### The Best Motives.

The producer might reasonably claim that the best motives are made to triumph, and that it conveys no objectionable moral. But the melodrama, which to an intelligent adult might well have an entirely comedy effect, cannot be good for the young. It creates morbid excitement or terror, which may disturb many a child's pillow, and while it is no more possible to exclude melodrama from the pictures than from the stage, there should be certain limits observed in a place which is so particularly the children's resort as the cinema. The opportunities of the cinema are so great, its technical achievements so remarkable, and a large proportion of its productions so altogether admirable, that it should not be difficult—even on purely commercial considerations—to eliminate crudities such as those indicated. They are sufficient to ruin the nerves of a sensitive child, and to give false values to all children who see them.

